# The spatial epidemiology and clinical features of reported cases of La Crosse Virus infection in West Virginia from 2003 to 2007

**DOI:** 10.1186/1471-2334-11-29

**Published:** 2011-01-26

**Authors:** Andrew D Haddow, Danae Bixler, Agricola Odoi

**Affiliations:** 1 The University of Tennessee, Department of Entomology & Plant Pathology, Knoxville, TN 37996-4560, USA; 2 The University of Texas Medical Branch, Department of Pathology and the Center for Biodefense and Emerging Infectious Diseases, 301 University Blvd. Galveston, Texas 77555-0609, USA; 3 West Virginia Bureau for Public Health, Room 125, 350 Capitol St. Charleston, WV 25301, USA; 4 The University of Tennessee, Department of Comparative Medicine, College of Veterinary Medicine 2407 River Dr. Knoxville, TN 37996-4543, USA

## Abstract

**Background:**

La Crosse virus (LACV) is a major cause of pediatric encephalitis in the United States. Since the mid-1980s, the number of reported cases of LACV infection in West Virginia has continued to rise and the state currently reports the most cases in the United States. The purpose of this study was to investigate and describe the spatial epidemiology and clinical presentation of LACV infection cases reported in West Virginia, as well as to provide a description of the environmental conditions present at the residences of the LACV infection cases.

**Methods:**

Descriptive and spatial analyses were performed on LACV infection cases reported to the West Virginia Department of Health from 2003 to 2007. Clinical and environmental variables were available for 96 cases and residence data were available for 68 of these cases. Spatial analyses using the global Moran's I and Kulldorff's spatial scan statistic were performed using the population 15 years and younger at both the county and census tract levels to identify those geographic areas at the highest risk of infection.

**Results:**

Two statistically significant (p < 0.05) high-risk clusters, involving six counties, were detected at the county level. At the census tract level, one statistically significant high-risk cluster involving 41 census tracts spanning over six counties was identified. The county level cumulative incidence for those counties in the primary high-risk cluster ranged from 100.0 to 189.0 cases per 100,000 persons (median 189.0) and the census tract level cumulative incidence for those counties in the high-risk cluster ranged from 61.7 to 505.9 cases per 100,000 persons (median 99.0). The counties and census tracts within high-risk clusters had a relative risk four to nine times higher when compared to those areas not contained within high-risk clusters. The majority of LACV infection cases were reported during the summer months in children 15 years and younger. Fever, vomiting, photophobia, and nausea were the most commonly reported signs and symptoms. A case fatality rate (CFR) of 3.1% was observed. Wooded areas and containers were present at the majority of case residences.

**Conclusions:**

The cumulative incidences of LACV infection from 2003 to 2007 were considerably higher than previously reported for West Virginia, and statistically significant high-risk clusters for LACV infection were detected at both the county and census tract levels. The finding of a high CFR and the identification of those areas at highest risk for infection will be useful for guiding future research and intervention efforts.

## Background

La Crosse virus (LACV), a member of the genus *Orthobunyavirus*, family *Bunyaviridae*, was first isolated from the brain of a pediatric patient who died of encephalitis in 1964 [1]. Since that time LACV has been recognized as one of the most common causes of pediatric arboviral encephalitis in the United States [2,3], with the majority of cases being reported in children under 15 years of age [4-8]. There are an average of 79 reported cases per year nationally [8], however the true incidence of LACV infections remains unknown, as most are undiagnosed and/or underreported [8,9]. The virus is transmitted to humans through the bite of infective mosquitoes, the primary vector being the eastern tree-hole mosquito, *Aedes triseriatus *[10], though two invasive species, the Asian tiger mosquito, *Ae. albopictus*, and the Asian bush mosquito, *Ae. japonicus*, have been incriminated as possible secondary or bridge vectors and both species are known to feed on humans [11-16]. La Crosse virus is maintained in a complex cycle involving both horizontal and vertical transmission in mosquitoes, as well as amplification in sciurid hosts [17-21], and is typically associated with areas of dense vegetation and/or stands of hardwood trees where the presence of vector species overlaps that of the amplification hosts [22-24]. The traditionally reported focus of virus transmission to humans has been the upper-Midwestern United States [2,4,5], but more recently LACV has been considered a major cause of pediatric encephalitic disease in West Virginia. Notably, West Virginia has led the nation in the number of annually reported cases since 1994 [8,25,26] (CDC unpublished).

From 1964 to 1986, there were 15 reported cases of LACV infection in West Virginia, comprising 0.9% of the nationally reported cases. Then in 1987, a pediatric referral center located in Charleston, West Virginia reported several cases of LACV infection. These findings led to active hospital-based surveillance for LACV infection cases in 15 West Virginian counties, ultimately resulting in the detection of 19 additional cases [27]. This case series marked a substantial increase in the number of reported LACV infection cases in the state and led to increased surveillance and research efforts. Following this outbreak, from 1987 to 2007, West Virginia reported 555 cases of La Crosse encephalitis to the Centers for Disease Control and Prevention (CDC), accounting for 31.4% of the total cases reported in the United States (CDC, unpublished data). A matched case-control study performed following the 1987 outbreak of LACV in West Virginia found a slight increase in disease risk for an increased time spent outdoors, the non-use of insect repellent, the non-use of air conditioning, a lack of screened windows and the non-use of protective clothing, while the presence of tree holes (natural water receptacles) near a residence was found to significantly increase the risk of virus transmission [28].

We conducted spatial and descriptive analyses of cases of LACV infection reported in West Virginia from 2003 to 2007 to determine those geographic areas at the highest risk for human infection and to assess both the clinical presentation of cases and the environmental conditions present at case residences.

## Methods

### Case data

Probable and confirmed LACV infection case data were collected through a passive surveillance system from 2003 to 2007, by the West Virginia Department of Health and Human Resources. Clinical data were available for 96 cases ranging in age from 0.4 years to 54.0 years (78 confirmed and 18 probable cases). Of these cases, 81 were 15 years or younger, of which 68 had data available on the location of their primary residence. The location of the primary residence for each case was determined by a geographic positioning system (GPS) reading taken at each case residence by the West Virginia Department of Health and Human Resources. Personal identifiers of cases were deleted before the data were released for the study. This research was exempted from the Institutional Review Board (IRB) review and certification under section 45 CFR 46.101(b) item 4 of the University of Tennessee's categories for exempted review involving the use of human subjects following a review by the Departmental Review Committee. The above item states that a research study may be exempt from IRB review if the research involves "the collection of or study of existing data, documents, records, pathological specimens, if these sources are publicly available or if the information is recorded by the investigator in such a way that subjects cannot be identified, directly or through identifiers linked to the subjects."

### Case definition

Confirmed cases of LACV infection are required to meet both the clinical and laboratory requirements set by the CDC case definition for neuroinvasive domestic arboviral diseases [29]. This definition is reprinted below:

In the absence of a more likely clinical explanation as documented by a physician, confirmed cases must meet all of the following criteria:

#### Clinical criteria

1) Fever, AND 2) Acutely altered mental status, or other acute signs of central or peripheral neurologic dysfunction, or pleocytosis associated with illness clinically compatible with meningitis,

AND

#### Laboratory criteria

3) A four-fold or greater change in virus-specific serum antibody titer, or isolation of virus from or demonstration of specific viral antigen or genomic sequences in tissue, blood, CSF, or other body fluid, or virus-specific immunoglobulin M (IgM) antibodies demonstrated in CSF by antibody-capture enzyme immunoassay (EIA), or virus-specific IgM antibodies demonstrated in serum by antibody-capture EIA and confirmed by demonstration of virus-specific serum immunoglobulin G (IgG) antibodies.

#### Probable case criteria

Cases that met the clinical definition and had stable (less than or equal to a two-fold change) but elevated titer of virus-specific serum antibodies, or virus-specific serum IgM antibodies detected by antibody capture EIA but with no available results of a confirmatory test for virus-specific serum IgG antibodies in the same or a later specimen, are deemed probable.

### Population, geographic, and environmental data

As the majority of LACV infections are pediatric [2,6,7], it was deemed appropriate to use the population 15 years and younger for the calculation of cumulative incidence for this study. The decennial 2000 United States Census was used to calculate the population 15 years and younger for each county and census tract in West Virginia. Counties are administrative and statistical subdivisions of a state. Census tracts are subdivisions of a county typically containing between 2,500 and 8,000 persons and are relatively homogeneous with respect to population characteristics, economic status and living conditions. Geographic boundary files were downloaded from the United States Census, TIGER, Geodatabase [30], and were used for all cartographic displays. Environmental officers of the West Virginia Department of Health and Human Resources conducted environmental assessments at the primary residence of both probable and confirmed cases' of LACV infection during the study period.

### Statistical and geographic analyses

Cumulative incidences were calculated at both the county and the census tract levels, and spatial analyses were performed on 68 cases 15 years and younger for which location data were available. Cumulative incidences were calculated for all counties in the study area (n = 55) and for counties reporting cases (n = 18), as well as for all census tracts in the study area (n = 466) and for census tracts reporting cases (n = 50). Cumulative incidence was expressed as the number of cases per 100,000 persons for the study period.

Descriptive analyses and the calculation of cumulative incidences were performed using STATA 10.0 [31]. The non-homogeneity of variances and resulting autocorrelation were adjusted for by smoothing the risk using spatial empirical Bayesian (SEB) smoothing [8,32-36]. This technique was implemented in GeoDa [37] using inverse distance spatial weights.

### Detection of Spatial clusters

The global Moran's I, implemented in GeoDa [37], was used to assess the presence of significant spatial autocorrelation of the unsmoothed cumulative incidences. Detection of spatial clusters of LACV infection were performed using Kulldorff's spatial scan statistic [38], and implemented in SaTScan [39]. A Poisson probability model was used to scan for geographical areas (counties and census tracts) with statistically significant high rates of LACV infections. A maximum spatial cluster size of 10% of the population 15 years and younger was used. For statistical inference, 9999 Monte Carlo replications were performed. The null hypothesis of no clusters was rejected when the simulated p ≤ 0.05. Cartographic displays were made using ArcView GIS 9.2 [40].

## Results

### Spatial analyses

The highest cumulative incidences for LACV infection cases were observed at the census tract level (Table 1). The cumulative incidence for counties reporting cases ranged from 7.2 to 166.8 per 100,000 persons (median 32.1), and from 42.4 to 505.9 per 100,000 persons (median 150.0) for those census tracts reporting cases.

**Table 1:** Comparisons of the Cumulative Incidence of Reported La Crosse Virus Infection Cases in the Population 15 Years and Younger for West Virginia County and Census Tracts, 2003 to 2007

			Cumulative incidence per 100,000 persons	
			
Geographic Risk Level		Obs.	Median	Range
Entire Study Area	County	55	0.0	0.0 - 166.8
	Census tract	466	0.0	0.0 - 505.9
Reporting Cases	County	18	32.1	7.2 - 166.8
	Census tract	50	150.0	42.4 - 505.9
High-Risk Clusters	County	4	83.2	40.2 - 166.8
	Census tract	30	156.5	61.7 - 505.9

Obs. = Observations, and refer to the number of counties or census tracts used for each analysis.

**Figure 1:**
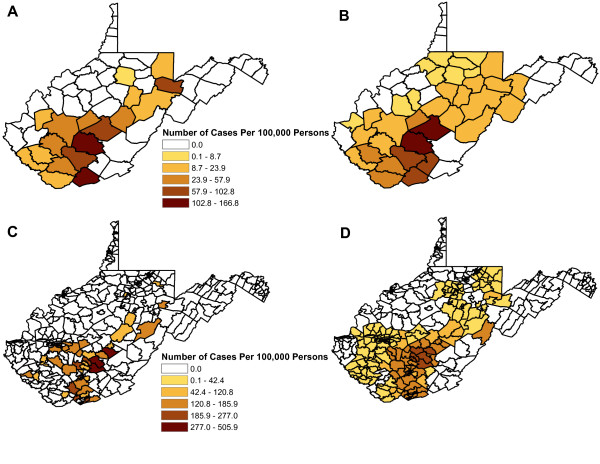
**The unsmoothed and smoothed cumulative incidence of La Crosse virus infections at the county and census tract levels in children 15 years and younger**. The distribution of unsmoothed risk of La Crosse virus infections at the county (A) and the census tract levels (C) for West Virginia. The distribution of spatial empirical Bayesian smoothed risk for La Crosse virus infections in West Virginia at the county (B) and the census tract levels (D).

The highest unsmoothed risks were observed in the south-central region of the state at both the county and census tract levels (Figure 1). Visually, the spatial patterns of SEB smoothed risks at the county and census tract levels were more evident and followed patterns similar to those of the unsmoothed risks (Figure 1), though only some of the census tracts within the high-risk counties had high-risks of infection.

**Figure 2:**
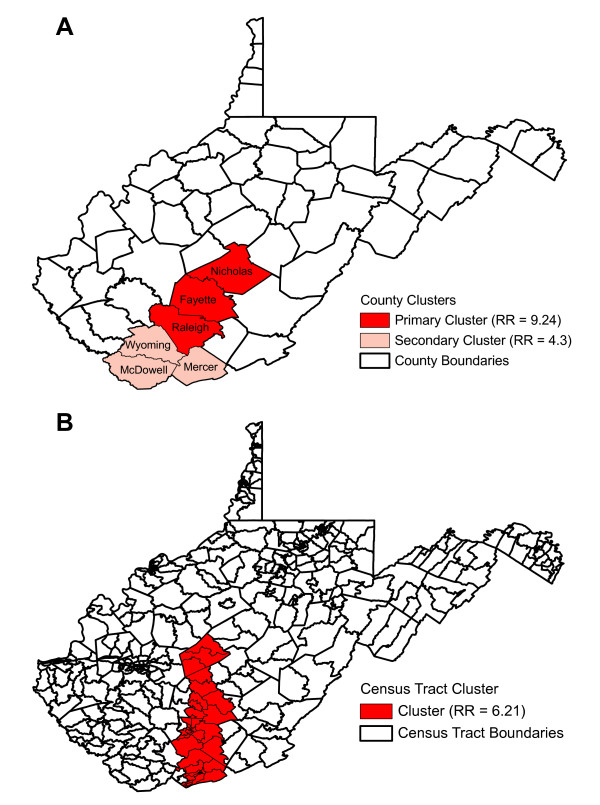
**Spatial clustering of La Crosse virus infection risk at the county and census tract levels in children 15 years and younger**. These maps show the significant high-risk clusters for La Crosse virus infection in West Virginia at the county (A) and at the census tract levels (B) detected by Kulldorff's Spatial Scan Statistic. RR = relative risk.

Statistically significant global clustering was detected at the county and census tracts with global Moran's I values of 0.4986 (p = 0.0001) and 0.2935 (p = 0.0001), respectively. Similarly, statistically significant local clusters (p < 0.05) of high-risk were detected at both the county and the census tract levels (Figure 2). At the county level, two statistically significant high-risk clusters were identified. The primary statistically significant (p = 0.0001) high-risk cluster consisted of three counties (Fayette, Raleigh, Nicholas). This cluster had a cumulative incidence ranging from 100 to 189 cases per 100,000 persons (median 111) and a relative risk of 9.2 implying that the risk of LACV infection was 9.2 times higher in these three counties than in those counties in the rest of the state of West Virginia. A secondary statistically significant (p = 0.0012) high-risk cluster was identified and included Wyoming, McDowell and Mercer counties. This cluster had a cumulative incidence ranging from 19 to 111 cases per 100,000 persons (median 40) and a relative risk of 4.3 (Figure 2) implying that the risk of LACV infection in this cluster was 4.3 times higher than that of those counties in the rest of the state. At the census tract level, one statistically significant (p = 0.0001) high-risk cluster, consisting of 41 census tracts, was identified. This cluster had a cumulative incidence ranging from 61 to 506 cases per 100,000 persons (median 99.0) and a relative risk of 6.2, implying that the risk of infection was 6.2 times higher than those census tracts not contained in the cluster. These census tracts were located in the following counties: Clay (3 census tracts), Fayette (8 census tracts), Mercer (14 census tracts), Raleigh (13 census tracts), Nicholas (1 census tract), and Summers (2 census tracts) (Figure 2).

### Clinical features

Cases of LACV infection ranged in age from 0.4 to 54.0 years, with a mean age of 10.8 (SD 10.3) years, of which 84.4% occurred in children 15 years and younger (Table 2). The vast majority of cases presented during July (28.1%), August (37.5%), and September (16.6%) in the southern region of the state. The most commonly reported signs and symptoms in cases were fever (76.0%), vomiting (75.0%), photophobia (52.1%), and nausea (50.0%) (Table 2). Other reported signs and symptoms included weakness (41.6%), stiff neck and confusion (33.3%), seizures (24.0%), and coma (4.2%). Meningitis and encephalitis were reported in 41.6% and 40.6% of the cases, respectively. A CFR of 3.1% was observed.

**Table 2:** Characteristics of Reported La Crosse Virus Infection Cases in West Virginia, 2003 to 2007

Variable	Number (%)
Sex	
Male	58 (60.4)
Female	38 (39.6)
Age	
0.4 - 0.9 yrs	1 (1.0)
1 - 5 yrs	27 (28.1)
6 - 10 yrs	38 (39.6)
11 - 15 yrs	15 (15.6)
16 - 20 yrs	6 (6.3)
≥ 21 yrs	9 (9.4)
Month of presentation	
May	1 (1.0)
June	8 (8.3)
July	27 (28.1)
August	36 (37.5)
September	16 (16.6)
October	8 (8.3)
Reported Signs and Symptoms	
Fever	73 (76.0)
Vomiting	72 (75.0)
Elevated CSF WBC	68 (70.8)
Photophobia	50 (52.1)
Nausea	48 (50.0)
Weakness	40 (41.6)
Meningitis	39 (40.6)
Encephalitis	38 (39.6)
Stiff Neck	32 (33.3)
Confusion	32 (33.3)
Seizures	23 (24.0)
Elevated CSF protein	24 (25.0)
Myalgia	11 (11.5)
Rash	7 (7.3)
Arthralgia	7 (7.3)
Coma	4 (4.2)
Died	
Male	2 (2.1)
Female	1 (1.0)

Health care providers reported either the presence of elevated cerebral spinal fluid (CSF) white blood cell (WBC) counts and/or the determined the counts. Elevated CSF WBC counts were reported in 73 cases (76.0%), of which 68 cases had a mean CSF WBC count of 160.9 per mm^3 ^(range: 10 to 670 per mm^3^, SD: 153.8 per mm^3^). Health care providers also either reported the presence of elevated CSF protein levels and/or the CSF protein levels. Elevated CSF protein levels were reported in 24 cases (25.0%). Cerebral spinal fluid protein levels were only available for 27 cases, with a mean level of 64.4, mg/dl (range: 24 to 359 mg/dl, SD: 61.6 mg/dl). Fever was present in all cases for which temperature was reported (n = 54), with a mean value of 39.4°C (range: 38.3°C to 40.6°C, SD: 0.66°C).

### Environmental observations

The most commonly observed environmental variables at case residences were the presence of a wooded area (92.0%) and the presence of containers (70.5%), which provide suitable habitats for mosquito larval development (Table 3). The presence of standing water was observed at almost half of the case residences (49.4%) and the majority of case residences (76.7%) were located within 45.6 m (149.0 ft) of a wooded area (Table 4).

**Table 3:** The Observed and Reported Environmental Conditions Present at the Primary Residences of La Crosse Virus Infection Cases in West Virginia, 2003 to 2007

Variable	Present (%)	Missing Data
Containers*	60 (70.6)	11
Tires	33 (40.7)	15
Tarps	32 (41.6)	19
Other containers^†^	53 (63.1)	12
Standing water	41 (49.4)	13
Wooded area	81 (92.0)	8
Hardwood	68 (94.4)	24
Evergreen	35 (71.4)	47

*Containers were all containers including: other containers, tires, and tarps. These variables were cross-referenced with one another for each case to determine the presence of at least one variable at each case site.

^†^Other containers were those containers not including tires and/or tarps.

**Table 4:** The Numbers of Observed and Reported Potential Larval Habitats Present at the Primary Residences of La Crosse Virus Infection Cases in West Virginia, 2003 to 2007

Variable	Number (%)	Missing Data
No. of tires		20
0	49 (64.5)	
1 - 9	21 (27.6)	
≥ 10	7 (9.2)	
No. of tarps		31
0	46 (70.8)	
1 - 10	21 (32.3)	
No. of other types of Containers*		23
0	19 (26.0)	
1 - 5	29 (39.7)	
6 - 15	10 (13.7)	
≥ 16	3 (4.1)	
Distance (meters) from residence to wooded area		23
0 - 14.9 (0 - 49 ft)	35 (47.9)	
15 - 45.6 (50 -149 ft)	21 (28.8)	
≥ 45.7 (≥ 150 ft)	6 (8.2)	

*Other types of containers were those containers not including tires and/or tarps.

## Discussion

Traditionally the highest number of reported LACV infection cases came from the upper-Midwestern states, but during the last 20 years the geographic area with the highest number of reported cases shifted to the Appalachian region of the United States [8]. Within this region, West Virginia now leads the nation in the number of reported cases annually [8]. To investigate the epidemiology of LACV infections in West Virginia, we performed spatial and descriptive analyses of LACV infection cases reported to the West Virginia Department of Health and Human Resources from 2003 to 2007.

The results of this study found that the highest cumulative incidences of LACV infection were located in the south-central region of the state, similar to the results of previous studies [27,28]. Furthermore, both the unsmoothed and SEB smoothed cumulative incidences were the highest in this region at both the county and census tract levels. To determine those areas at the highest risk for disease, we performed spatial analyses at two different spatial levels (county and census tract). The census tract level was used in this study over a smaller geographic level (i.e., census block) because this is typically the smallest spatial level that is acceptable for the public reporting of diseases under the Health Insurance Portability and Accountability Act (HIPAA) and due to the availability of census data variables during the study period. The high cumulative incidences observed in this study were similar to those high cumulative incidences reported in eastern Tennessee [36], the location of another area of high-risk LACV infection case clustering in the Appalachian region [8,36]. Significant high-risk clustering was observed at both the county and census tract levels in the south-central region of West Virginia. Two statistically significant clusters of high LACV infection risk (comprising six counties) were detected at the county level with a relative risk roughly four to nine fold higher than those counties located outside of the high-risk clusters, and one statistically significant cluster (comprising 41 census tracts within six counties) was observed at the census tract level with a relative risk about six times higher compared to those census tracts located outside of the high-risk cluster. These areas of high-risk should form the focus of education and intervention efforts in the future. Additionally, the results of this study reaffirm that the census tract level is the preferred geographic level for reporting cases and conducting analyses of focal diseases [36,41].

In agreement with previous work [7,8], our study found that the majority of reported LACV infections occur in male children 15 years and younger during the summer months, and that cases display fever, headache, vomiting, and mental status changes. La Crosse virus infection has traditionally proven difficult to distinguish from herpes simplex meningoencephalitis [7,42]. Our study demonstrates that elevated CSF WBCs remain a hallmark of LACV infection, however it should be noted that patients frequently demonstrate a predominance of polymorphonuclear leukocytes on their peripheral blood smears and in their CSF, which is suggestive of a bacterial infection and may result in a delayed diagnosis of LACV infection [7]. A troubling discovery in this study was the continued high CFR (3.1%) in West Virginia [28], compared to a lower CFR (1.5%) reported during the same time period for the rest of the United States [8]. The reason for this higher CFR in West Virginia is unclear, but may indicate the possibility of a more virulent strain(s) of the virus circulating in this region [43,44], reporting bias, clinical management, and/or chance variation. The reasons underlying the increased CFR warrant further investigation.

The reason for the increase in the number of reported cases of LACV infection in the Appalachia region and in West Virginia, in the past 20 years remains unknown. One possibility is a change in the geographic distribution of vector species, and recent work has indicated a possible geographic shift in vector abundance between *Ae. triseriatus*, *Ae. albopictus*, and *Ae. japonicus *populations in West Virginia [45-47]. Of interest, the number of reported cases of LACV infection in West Virginia has decreased in recent years (2005 to 2009) when compared to data from the previous decade (CDC unpublished). Prior to the establishment of *Ae. albopictus *and *Ae. japonicus *in West Virginia, LACV had been isolated from *Ae. triseriatus *mosquitoes collected in 1996 at former sites of confirmed LACV infection cases in West Virginia [26]. A recent survey of abandoned tires to determine the presence of mosquito larvae in western, central and eastern West Virginia found that *Ae. japonicus *was the most frequently collected larval mosquito species throughout the state in this habitat type. Furthermore, their survey revealed that *Ae. albopictus *was collected at significantly lower numbers than *Ae. triseriatus *at both peridomestic and non-peridomestic tire sites in the central region of the state [45], where the results of this study indicate the highest disease risk. However, a study in eastern Tennessee collecting adult host-seeking mosquitoes using CO_2 _baited CDC traps from 2004 to 2006 found that *Ae. albopictus *was three times more likely to be collected than *Ae. triseriatus *at the residences of previously confirmed LACV cases, in addition to residences without a prior history of reported LACV cases [14]. Similar results were observed at active LACV case residences using human landing collections in 2004 [14].

The observation that a large number of case residences in this study were in close proximity to wooded areas and had the presence of containers are in agreement with those findings of a case-control study previously performed in this region which found them to be risk factors for LACV infection in West Virginia [28]. Such characteristics provide a habitat for vector species and amplification hosts [20-24]. Thus, as observed elsewhere, the closure of tree-holes and the elimination of water-holding containers would likely reduce the overall burden of infective mosquitoes and decrease the risk of LACV transmission [48]. Importantly, although LACV has not been isolated from mosquitoes in West Virginia since 1996, the lack of isolates are likely the result of extremely limited testing of mosquito pools for LACV rather than a true lack of virus presence in mosquito populations. Ultimately, the status of *Ae. triseriatus*, *Ae. albopictus*, and *Ae. japonicus *concerning the transmission and maintenance of LACV in West Virginia remains unknown, and research is urgently needed to fill this gap in our understanding of the epidemiology of LACV.

## Limitations

Due to the methodology and data employed in our study there are some limitations. Passive surveillance systems are prone to under-reporting/detection of disease, though we feel that the majority of cases suffering from severe illness were diagnosed and reported to state health officials. Clinical data were collected from multiple health care providers by local health department personnel for the purpose of case ascertainment. Due to limited resources, verification of complete and accurate reporting of laboratory and clinical features was not possible. As data regarding the clinical features of cases of LACV infection were extracted from patient charts and/or recalled by healthcare professionals post-presentation of disease, the actual frequency of reported signs and symptoms could be higher than those reported in this study. Missing information for environmental data resulted when visits to the cases' residences were not possible. The environmental information presented in this paper is purely for descriptive purposes and is not intended to infer an association with LACV infections.

## Conclusions

During the study period, West Virginia reported the highest cumulative incidence of LACV infection cases in the United States, marking a shift from the traditional geographic focus of reported LACV infection cases from the upper-Midwestern states to the Appalachian region [8]. The reason for this shift is unclear, but could be due to a variety of factors including increases and/or decreases in diagnosis, reporting, education, and prevention. Additionally, changes in virus strain virulence, the distribution of vector species, land use patterns, forest succession and/or increased human encroachment into enzootic foci could also be contributing factors. Future surveillance efforts should include both the screening of vector species for virus, as well as determining the seroprevalence to LACV in amplification hosts. In addition, LACV isolations are needed so West Virginian virus strains can be compared with virus strains from other regions to determine if differences exist in virulence and vector competence. Although the reporting of the clinical presentation of cases of LACV infection through a passive surveillance system is not without limitations, such reporting will allow for the continued monitoring of infection patterns. The findings of this study demonstrate that south-central West Virginia remains a focus of LACV transmission and highlights the utility of using the combination of cumulative incidence, relative risk, and spatial statistics to determine those geographic areas at highest risk for infection. The majority of reported LACV infection cases during the study period had the presence of wooded areas and containers near their primary residence corroborating the results of a previous case-control study. Finally, the results of this study will be useful for guiding disease control strategies and will allow public health officials to target specific areas for interventions.

## Competing interests

The authors declare that they have no competing interests.

## Authors' contributions

ADH conceived and designed the study, performed the experiments, and wrote the manuscript. DB contributed the data and participated in helping draft the manuscript. AO helped design the study, perform the experiments, and participated in helping draft the manuscript. All authors read and approved the final manuscript.

## Pre-publication history

The pre-publication history for this paper can be accessed here:

http://www.biomedcentral.com/1471-2334/11/29/prepub
